# Increased proteasomal activity supports photoreceptor survival in inherited retinal degeneration

**DOI:** 10.1038/s41467-018-04117-8

**Published:** 2018-04-30

**Authors:** Ekaterina S. Lobanova, Stella Finkelstein, Jing Li, Amanda M. Travis, Ying Hao, Mikael Klingeborn, Nikolai P. Skiba, Raymond J. Deshaies, Vadim Y. Arshavsky

**Affiliations:** 1 Department of Ophthalmology, Duke University School of Medicine, Durham, NC 27510 USA; 2 Division of Biology and Biological Engineering, California Institute of Technology, Pasadena, CA 91125 USA; 3 Department of Pharmacology and Cancer Biology, Duke University School of Medicine, Durham, NC 27510 USA; 4 Howard Hughes Medical Institute, California Institute of Technology, Pasadena, CA 91125 USA; 5 Present Address: Department of Ophthalmology, University of Florida, Gainesville, FL 32610 USA; 6 Present Address: Amgen, One Amgen Center Way, Thousand Oaks, CA 91320 USA

## Abstract

Inherited retinal degenerations, affecting more than 2 million people worldwide, are caused by mutations in over 200 genes. This suggests that the most efficient therapeutic strategies would be mutation independent, i.e., targeting common pathological conditions arising from many disease-causing mutations. Previous studies revealed that one such condition is an insufficiency of the ubiquitin–proteasome system to process misfolded or mistargeted proteins in affected photoreceptor cells. We now report that retinal degeneration in mice can be significantly delayed by increasing photoreceptor proteasomal activity. The largest effect is observed upon overexpression of the 11S proteasome cap subunit, PA28α, which enhanced ubiquitin-independent protein degradation in photoreceptors. Applying this strategy to mice bearing one copy of the P23H rhodopsin mutant, a mutation frequently encountered in human patients, quadruples the number of surviving photoreceptors in the inferior retina of 6-month-old mice. This striking therapeutic effect demonstrates that proteasomes are an attractive target for fighting inherited blindness.

## Abstract (web summary)

Proteasomal overload can be found in a broad spectrum of mouse models of retinal degeneration. Here the authors find that overexpressing the PA28α subunit of the 11S proteasome cap increased the number of surviving functional photoreceptor cells in a mouse model of retinal degeneration bearing the P23H mutation in rhodopsin.

## Introduction

Hereditary degenerative diseases of the retina, including retinitis pigmentosa (RP), affect nearly 2 million patients worldwide^1^. These conditions are caused by ~4500 distinct mutations in more than 250 genes^2^. Such incredible genetic diversity complicates the understanding of underlying pathology and suggests that the most useful therapeutic interventions would employ mutation-independent strategies that can ameliorate the cellular pathology shared across large groups of mutations and patients. Studies of animal RP models have revealed a number of common pathological conditions: oxidative stress^3^, unfolded protein response^4,5^, retinoid cytotoxicity^6^, iron toxicity^7^, and aberrant phototransduction^8^.

Our recent work demonstrated that another major cellular stress factor prevalent in a broad spectrum of mouse RP models is the insufficient capacity of the ubiquitin–proteasome system to process misfolded or mistargeted proteins in affected cells^9^. We further demonstrated that the severity of photoreceptor retinal degeneration correlates with the degree of misfolded protein production. A similar condition has subsequently been found in the mouse model of Bardet–Biedl Syndrome^10^, a disorder affecting photoreceptors among other ciliated cells^11^. Conversely, genetic manipulation reducing the proteolytic capacity of proteasomes evoked RP-like pathology in otherwise normal retinas^12^.

The goal of the present study was to determine whether survival of degenerating photoreceptors could be supported by enhancing the proteolytic capacity of their proteasomes. We aimed to increase the proteasome activity in these cells using two independent genetic strategies and found that the strongest effect was achieved by overexpressing the PA28α subunit of the 11S proteasome cap. We also show that the underlying mechanism is based primarily on the stimulation of ubiquitin-independent protein degradation. Breeding PA28α-overexpressing mice with two mouse models of photoreceptor degeneration results in a delay of disease progression. Particularly striking photoreceptor preservation is observed in the mouse bearing one copy of the P23H mutation in rhodopsin gene, which is frequently encountered in North American RP patients.

## Results

### Proteasomal composition of the mouse retina

To develop efficient strategies for enhancing proteasomal activity in photoreceptors, we first analyzed the molecular composition of proteasomes in the mouse retina. Proteasomes are multi-subunit complexes assembled upon association of two principal components: the 20S core and the regulatory caps. The 20S core is responsible for the entire proteolytic process; however, its basal activity is very low in the absence of regulatory caps. The most common caps are 19S and 11S, which facilitate protein degradation in ubiquitin-dependent and -independent manners, respectively. A single 20S core may associate with one or two caps (either identical or different) at the sites located at the opposite ends of the 20S barrel-like structure^13,14^.

**Fig. 1:**
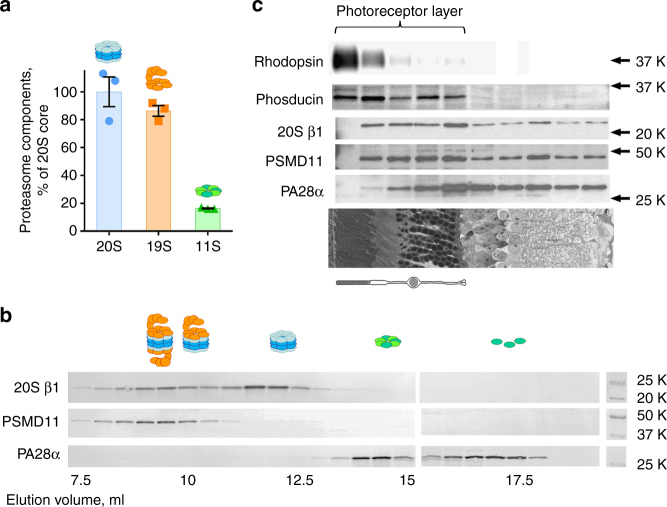
Proteasome composition of the mouse retina. **a** The molar ratio among 20S, 19S, and 11S proteasomal components determined by quantitative mass spectrometry. Data are shown as mean ± SEM; *n* = 3. **b** Fractionation of proteasome components in retinal extracts from 1-month-old mice (200 μg total protein) by size-exclusion chromatography on a Superose-6 column. Proteins in 0.5 ml fractions were probed by western blotting using antibodies against β1 subunit of the 20S proteasome core, PSMD11 subunit of the 19S proteasome cap, and PA28α subunit of the 11S cap. Data are taken from one of the four similar experiments. **c** The distribution of β1, PSMD11, and PA28α in 20 μm serial tangential sections throughout the entire WT mouse retina. Each section was solubilized in 30 μl SDS-PAGE sample buffer for analysis. Proteins were visualized by western blotting using the ECL technique. Rhodopsin was used as a photoreceptor outer segment marker; phosducin was used as a marker of the entire photoreceptor layer. Data are taken from one of two similar experiments. A representative retinal cross-section is shown below western blot panes; the corresponding position of the photoreceptor cells is illustrated by a cartoon

We first determined the stoichiometry among these three proteasomal components in the whole wild-type (WT) mouse retina using quantitative mass spectrometry with isotope-labeled peptide standards corresponding to representative proteasome subunits: α7 and β1 for 20S, PSMD5 and PSMD6 for 19S, and PA28α for 11S (Fig. 1a; see Methods for details of this analysis). These measurements revealed that the amount of 19S caps in the mouse retina is equivalent to 86 ± 7% of 20S cores, while the amount of 11S caps corresponds to only 16 ± 1% of 20S cores (taking into account that each 11S particle contains seven PA28 subunits represented by roughly equal amounts of PA28α and PA28β) (mean ± SEM; *n* = 3).

We then assessed the patterns in which these proteasomal components associate with one another. Retinal extracts obtained from WT mice were subjected to size-exclusion chromatography and the distribution of proteasomal complexes across chromatographic fractions was determined by western blotting of proteins representing each of the 20S, 19S, and 11S particles (β1, PSMD11, and PA28α, respectively; Fig. 1b). Experiments were performed in the presence of ATP, which is essential to preserve the 20S–19S complex assembly^15^. A typical chromatogram, shown in Fig. 1b, illustrates that 20S core particles eluted in two peaks of similar sizes. One, containing 45 ± 2% (mean ± SEM; *n* = 4) of 20S cores, co-eluted with the entire pool of 19S caps and another, containing 55 ± 2% of 20S, did not co-elute with either cap type. This excess of free 20S cores is a common phenomenon previously observed in various tissues and cell types^16–18^. Unlike 19S caps, 11S caps migrated in fractions lacking the 20S core, which could be explained either by the absence of 11S–20S complex in normal retinas or relatively low stability of this complex upon protein extraction and chromatography due to the relatively low affinity between 11S caps and 20S cores^19,20^. Additional evidence that the amount of 20S core particles in the retina exceeds the amount of 19S cap was obtained by performing native gel electrophoresis of retinal lysates (Supplementary Fig. 1). Whereas the entire pool of 19S caps was distributed between the bands representing singly and doubly capped 20S cores, 20S exhibited a third band corresponding to its non-capped form.

Next, we estimated the fraction of proteasomes residing in rod photoreceptor cells. The retina is a layered tissue consisting of many cell types connected in complex networks. This makes obtaining preparative quantities of pure photoreceptor cells a nearly impossible task. However, because (i) photoreceptor cells form a compact layer which occupies roughly one half of the retinal volume (as illustrated in a representative retina cross-section in Fig. 1c) and (ii) ~97% of mouse photoreceptors are rods^21^, the fractional content of any retinal protein confined to rods can be estimated by analyzing the prevalence of this protein in serial tangential sections representing the photoreceptor-dense half of the retina^22^. Therefore, we obtained serial tangential sections through the entire flat-mounted retina, measured the levels of proteasomal proteins across all individual sections by western blotting, and calculated their fractional contents in the photoreceptor layer, i.e., those containing the photoreceptor-specific protein marker, phosducin (Fig. 1c). This estimate revealed that rod photoreceptors contain ~2/3 of all retinal proteasomes (65 ± 7% of 20S, 60 ± 4% of 19S, and 47 ± 4% of 11S; *n* = 2).

Overall, these experiments demonstrated that photoreceptors contain more than one half of all retinal proteasomes, which are represented primarily by comparable amounts of 26S complexes and free 20S cores. This suggests that the simplest approach to enhance proteasomal activity in these cells would be to stimulate the activity of free 20S cores by increasing the amount or affinity of regulatory caps. We followed this strategy in subsequent experiments.

### Rod proteasome activity can be enhanced by PA28α overexpression

**Fig. 2:**
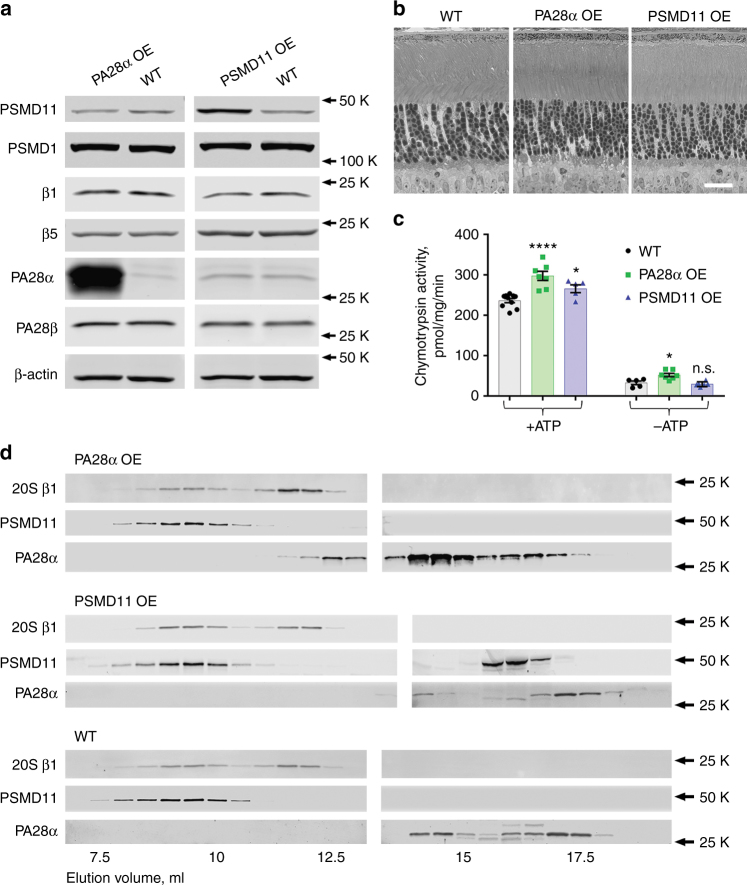
Characterization of PA28α and PSMD11 overexpressing (OE) mice. **a** Western blots of proteasomal subunits in retinal lysates containing 30 μg total protein. Bands were visualized using the LiCor Odyssey imaging system. Each protein was analyzed in at least 3 pairs of 1-month-old WT and overexpressing animals. **b** Retinal morphology of 3-month-old overexpressing and WT mice. Retinas were embedded in plastic, 1 μm cross-sections were stained by toluidine blue and analyzed by light microscopy. Data are taken from one of the five similar experiments; scale bar: 20 μm. **c** Chymotrypsin-like proteasomal activity in retinal extracts from 1-month-old overexpressing and WT mice; measurements were performed in the presence or absence of ATP, as indicated. The number of measurements was 10, 7, and 5 for WT, PA28α overexpressing, and PSMD11 overexpressing mice, respectively. The data are shown as mean ± SEM; *p* values determined across individual preparations are indicated in the text. **d** Fractionation of proteasomal components in retinal extracts from 2-month-old overexpressing and WT mice by size-exclusion chromatography on a Superose-6 Increase column. Proteins in 0.5 ml fractions were probed by western blotting using antibodies against the β1 subunit of the 20S proteasome core, PSMD11 subunit of the 19S proteasome cap, and PA28α subunit of the11S cap. Data are taken from one of the three similar experiments

Our first approach to enhance proteasome activity in rods was the overexpression of 11S caps, whose binding to the ends of 20S core particles facilitates substrate access to proteolytic activities confined inside the 20S barrel-like structure. Normally, this cap is formed by a hetero-heptameric ring consisting of PA28α and PA28β subunits^23^. The stoichiometry between PA28α and PA28β was assessed to be either 3:4 or 4:3 in two independent in vitro studies^24,25^. However, functional 11S caps can also be formed by the PA28α subunits alone, although they bind 20S cores with a lower affinity and stimulate their proteolytic activity to a lesser degree than hetero-heptameric caps^20,24^. Therefore, we overexpressed PA28α using the well-established transgenic protocol utilizing the rod-specific rhodopsin promoter^26^. Three mouse lines underwent germline transmission. Among them, we selected the one whose retinal PA28α content was 77±3-fold higher than in WT mice (mean ± SEM; *n* = 5; Fig. 2a; see Supplementary Fig. 2 for representative data). This corresponds to a comparable level of 11S cap overexpression in rods because the fraction of endogenous PA28α in rods and the fraction of PA28α in endogenous 11S caps are each close to 50% and, therefore, cancel one another in this calculation. Considering the ratio between the endogenous 20S and 11S particles in photoreceptors estimated above and assuming that all expressed PA28α subunits assemble into 11S caps, we conclude that the resulting 11S content in transgenic rods exceeds their 20S content by ~9-fold.

Western blot analysis of retinal extracts obtained from transgenic animals demonstrated that PA28α overexpression did not affect the expression of other proteasomal subunits (Fig. 2a). This differs from two previous reports that PA28α overexpression in cell culture and transgenic mouse cardiomyocytes causes a concurrent overexpression of PA28β^27,28^, likely reflecting variation in gene regulation patterns across individual cell types. Importantly, the overexpression of PA28α did not cause any adverse effects on photoreceptor health, as evidenced by normal photoreceptor morphology analyzed in 3-month-old transgenic mice (Fig. 2b).

The effect of PA28α overexpression on proteasomal activity in retinal extracts was first assessed using the fluorogenic substrate of chymotrypsin-like activity, Suc-LLVY-amc. The activity measured in extracts from overexpressing retinas in the presence of ATP was 26% higher than extracts from WT controls (*p* = 7×10^−5^; Fig. 2c). Given that rods contain close to 2/3 of total retinal proteasomes, this corresponds to ~40% activity increase in these cells. Enhanced proteolysis of the fluorogenic peptide substrate caused by PA28α overexpression was preserved in the absence of ATP (63% increase over WT; *p* = 0.01; Fig. 2c, right panel), although the absolute activity increase in this case was smaller than when ATP was present.

**Fig. 3:**
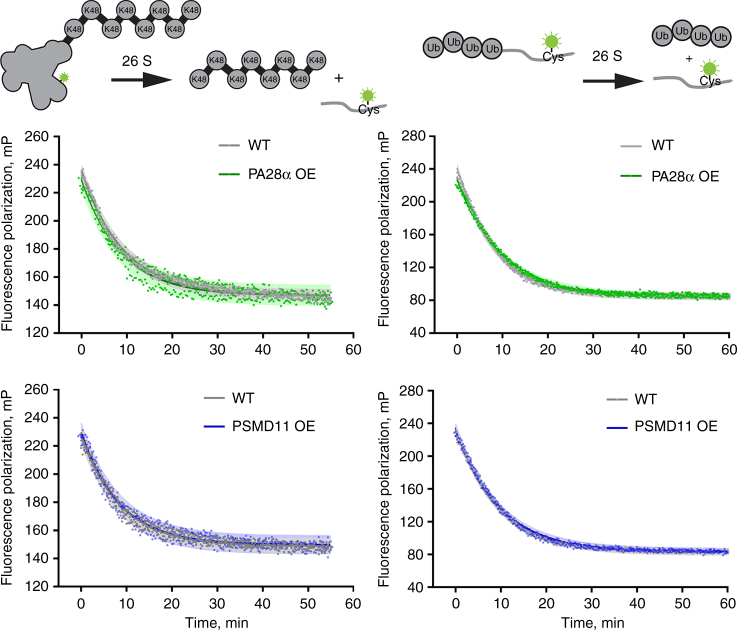
Overexpression of PA28α or PSMD11 does not affect ubiquitin-dependent proteasome activity. The decrease in fluorescence polarization representing the degradation of polyubiquitinated protein (left) or tetraubiquitinated peptide (right) substrates was monitored in retinal extracts from 1-month-old mice of indicated genotypes. Data points represent individual measurements collected every 20 s in three technical replicates performed with the same pair of retinal extracts. Data were fitted with single exponents (solid lines); shaded areas show 95% confidence intervals of the fits. The data are taken from one of the three similar independent experiments. In all cases, *R*^2^ values for exponential fits were at least 0.96 for the polyubiquitin and 0.99 for the peptide substrates

In contrast, overexpression of PA28α did not significantly affect the ubiquitin-dependent proteasome activity in retinal lysates. This lack of activity increase was observed in experiments conducted with both polyubiquitinated protein and a tetraubiquitin-peptide fusion (Fig. 3).

We next attempted to identify proteasome complexes formed upon PA28α overexpression. One possibility is that overexpressed 11S caps bind at one or both ends of free 20S core particles. Alternatively, 11S overexpression could lead to the formation of “hybrid proteasomes”, which contain a 19S cap at one end and an 11S cap at the other^29^. We conducted size-exclusion chromatography of retinal lysates from PA28α overexpressing mice and found that a distinct portion of PA28α elutes earlier than in control WT lysates, partially overlapping with 20S cores (Fig. 2d). Averaging data across three independent chromatography runs (Supplementary Fig. 3) showed that this portion of PA28α represents ~4% of its total amount and ~2% of PA28α co-elutes with 20S. This suggests that PA28α overexpression leads to formation of the 20S–11S complexes. However, the lack of complete overlap between the profiles of this leftward-shifted PA28α and the entire peak of 20S cores free of 19S suggests that these putative complexes are unstable and fall apart in the course of a chromatography run. This is consistent with the low affinity between 20S particles and 11S caps formed exclusively from the PA28α subunits^20,24^. Interestingly, we did not observe a notable reciprocal leftward shift in the chromatography profile of the second 20S peak upon PA28α overexpression. This may be explained by a combination of modest increase in the 20S’ Stokes radius upon binding a relatively compact 11S cap and the instability of this complex during a chromatography run. We also analyzed chromatographic behavior of the endogenous PA28β subunit and found no difference between WT and PA28α overexpressing lysates (Supplementary Fig. 3). Therefore, all 11S caps formed upon PA28α overexpression are homomeric. Finally, the overexpression of PA28α did not result in any fraction of the 19S caps eluting apart from the 20S cores, which argues against a hypothetical possibility that overexpressed 11S caps out-competed endogenous 19S caps from their 20S complexes.

In conclusion, PA28α overexpression appears to be a viable approach to enhancing proteasomal activity in rods, particularly in respect to the processing of non-ubiquitinated substrates.

### Rod proteasome activity can be enhanced by PSMD11 overexpression

The structure of the 19S proteasome cap is more complex than the 11S cap. It consists of 19 individual proteins forming two subassemblies^30^. Although we are not aware of evidence suggesting that overexpressing any of these proteins could increase the cellular content of 19S caps, several studies showed that proteasomal activity could be enhanced by overexpressing one of its subunits, PSMD11^31–34^. The underlying mechanism is not fully understood and is thought to involve stabilization of the 19S–20S complex^31^, likely requiring PSMD11 phosphorylation^33^. We overexpressed PSMD11 in rods using the same transgenic approach as above.

Of four mouse lines that underwent germline transmission, we selected the highest expressing line whose retinas contained 4.2 ± 0.1-fold more PSMD11 than control WT littermates (mean ± SEM; *n* = 4; Fig. 2a; see Supplementary Fig. 2 for representative data), corresponding to a roughly 6-fold PSMD11 overexpression in rods. Just as in the case of PA28α, PSMD11 overexpression did not adversely affect photoreceptor health (Fig. 2b). Proteasomal activity measured in retinal extracts from PSMD11 overexpressing mice using the fluorogenic (AMC)-conjugated peptide substrate, Suc-LLVY-amc, was 12% higher than in extracts from WT controls (*p* = 0.011; Fig. 2c), which corresponds to ~18% activity increase in rods (calculated as above). In this case, the activity increase was strictly ATP-dependent. Notably, this effect was smaller than the ~40% increase achieved upon PA28α overexpression (although the *p* value of 0.07 between the two overexpression conditions fell slightly short of reaching statistical significance).

We examined the ubiquitin-dependent proteasome activity in retinal extracts from PSMD11 overexpressing mice using both polyubiquitinated protein and a tetraubiquitin-peptide fusion substrate, but did not observe any notable deviation from the corresponding activities analyzed with their WT littermate controls (Fig. 3). This suggests that proteasomal activation in this case was at least mostly ubiquitin independent. Size-exclusion chromatography of retinal extracts from PSMD11 overexpressing mice yielded a profile very similar to WT extracts, except for a distinct PSMD11 portion eluting toward the end of the chromatographic profile, presumably not associated with any other proteins (Fig. 2d). Taken together, these data indicate that PSMD11 overexpression may serve as an approach to proteasomal activity enhancement in rods, although not as promising as PA28α overexpression.

### Proteasome activity enhancement improves the survival of P23H rods

In the next series of experiments, we tested whether proteasomal activity enhancement could ameliorate photoreceptor degeneration caused by an RP-associated mutation. We chose a mouse model bearing a P23H single amino acid substitution at one rhodopsin allele^35^, a mutation prevalent among North American RP patients^36^. The underlying pathology is thought to be associated with inability of mutant rhodopsin to properly fold in the endoplasmic reticulum (ER). Instead of being delivered to photoreceptor outer segments, P23H rhodopsin undergoes ER-associated degradation (ERAD), which targets misfolded proteins to the proteasomal degradation pathway^37^. This process ultimately evokes an unfolded protein response^37^, and compromises the capacity of the ubiquitin–proteasome system to process other cellular substrates, as documented in our previous study of an alternative mouse model expressing the P23H rhodopsin mutant transgene^9^. Interestingly, the degradation of unfolded P23H rhodopsin is so efficient that no prominent aggregates form at any stage of this pathology^35^.

We now confirmed that, like in transgenic P23H mice, rods of the P23H knockin mice suffer from proteasomal insufficiency (Supplementary Fig. 4). This is evident from the accumulation of the Ub^G76V^–GFP proteasomal activity reporter in mutant photoreceptors (panels A and B) and increased levels of polyubiquitinated proteins in the retina (panels C and D). At the same time, the amount and the activity of proteasomes in mutant retinas remained essentially unchanged (panels E and F, respectively). These parameters were measured in young P23H mice in order to minimize any complications associated with the ongoing photoreceptor loss.

**Fig. 4:**
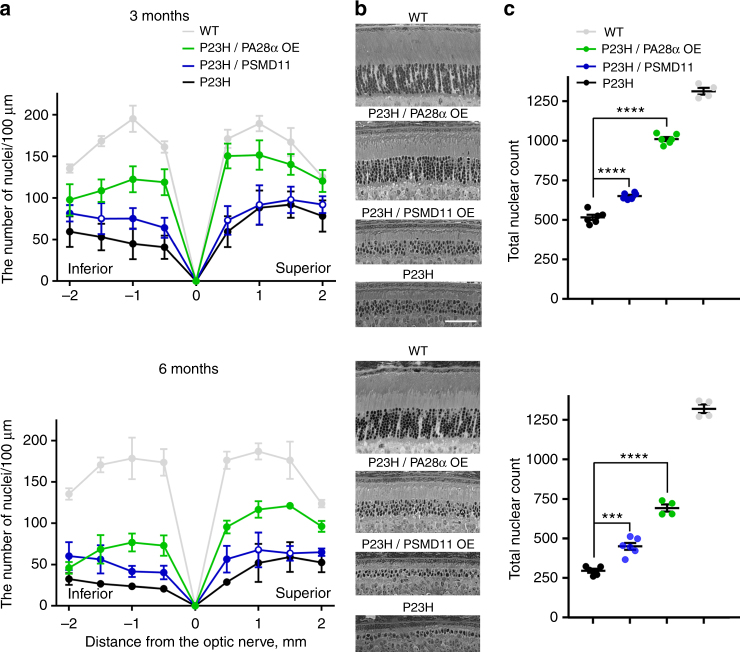
Overexpression of PA28α or PSMD11 improves photoreceptor survival in P23H mice. **a** Spider diagrams representing the number of photoreceptor nuclei in 100 μm segments of the inferior and superior retina counted at various distances from the optic nerve head. Data collected from 3- and 6-months-old mice are shown as mean ± SD. The number of eyes analyzed at 3 months was: P23H—6, P23H/PA28α OE—6, P23H/PSMD11 OE—6, WT—4; the number of mice analyzed at 6 months was: P23H—5, P23H/PA28α OE—4, P23H/PSMD11 OE—6, WT—4. Data points for which the difference in the number of nuclei between treated and untreated animals was statistically significant (*p* < 0.05) are shown as filled circles and those for which statistical significance was not achieved as open circles. **b** Representative images of inferior retina cross-sections at the 1 mm distance from the optic nerve head. Mouse genotypes are indicted above the panels. Scale bar: 50 μm. See Supplementary Figs. 6 and 7 for images of representative cross-sections through the entire retinas. **c** The total number of nuclei in all eight 100 μm retinal segments presented in the spider diagrams of **a**. Data are shown as mean ± SEM

P23H mice were crossed with mice overexpressing either PA28α or PSMD11. Measurements in retinal lysates conducted with the Suc-LLVY-amc substrate confirmed that the proteasomal activity increase caused by these proteins’ overexpression was essentially the same as in WT mice (Supplementary Fig. 5) The morphological phenotypes of these mice were analyzed in retinal cross-sections at the ages of 3 and 6 months (Fig. 4 and Supplementary Figs. 6 and 7). Even superficial examination of these sections shows that retinal degeneration was slowed in both cases, with overexpression of PA28α causing a particularly striking effect. To quantify this effect, we counted the number of photoreceptor nuclei in 100 μm retinal segments at different distances from the optic nerve and plotted the results as spider diagrams (Fig. 4a). These diagrams document substantial improvement in photoreceptor survival across the entire retina.

Of particular interest is the part of inferior retina adjacent to the optic nerve head, which undergoes the most rapid photoreceptor loss in this animal model^37^. At the age of 3 months, mice overexpressing PSMD11 retained ~40% more surviving photoreceptors than their WT littermates, as judged from the number of photoreceptor nuclei in this area, whereas overexpression of PA28α increased this number by a remarkable 130% (Fig. 4b). By the age of 6 months, the P23H retina was devastated, with a single row of remaining photoreceptor nuclei representing mostly surviving cones. In contrast, P23H mice overexpressing PSMD11 preserved at least one additional row of nuclei, while mice overexpressing PA28α had at least three additional rows.

To quantify these changes across the entire retina, we combined the nuclear counts obtained at all locations analyzed in the spidergrams and plotted the resulting values for each genotype and age (Fig. 4c). This analysis revealed that overexpression of PA28α increased photoreceptor survival by ~2 and ~2.3-fold at 3 and 6 months, respectively. The corresponding parameters observed upon PSMD11 overexpression were ~1.3 and ~1.5-fold.

To assess whether the preservation of photoreceptor morphology in these mice was in fact associated with improved retinal function, we analyzed them using electroretinography (ERG). ERGs are massed field potentials which reflect the combined light-evoked activity of many retinal cells. The negative ERG deflection immediately following the flash stimulus, called the a-wave, originates primarily from the suppression of the circulating dark current in rod and cone outer segments. The subsequent positive deflection, called the b-wave, originates mainly from the light-induced depolarizing currents in ON-bipolar cells downstream from photoreceptors and represents amplified photoreceptor responses^38,39^. In degenerating retinas, changes in the amount and health of surviving photoreceptors are reflected by reduced amplitudes of both waves.

**Fig. 5:**
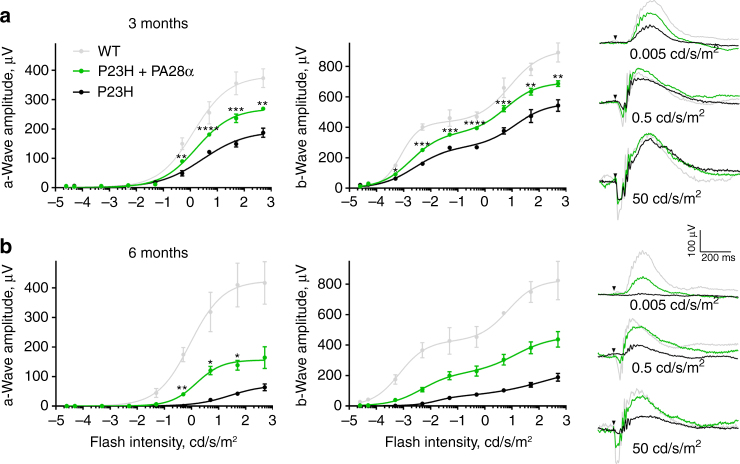
Overexpression of PA28α improves retinal function in P23H mice. **a**, **b** Response amplitudes of ERG a- and b-waves evoked by light flashes of increasing intensity were measured in PA28α overexpressing P23H mice, their control P23H littermates, and WT animals at the ages of 3 (**a**) and 6 (**b**) months. The number of eyes analyzed at 3 months was: P23H—6, P23H/PA28α OE—6, WT—3; the number of mice analyzed at 6 months was: P23H—3, P23H/PA28α OE—4, WT—4. Data were averaged and fitted using a single (for the a-wave) or double (for the b-wave) hyperbolic function. Error bars represent SEM. On the right are shown representative ERG recordings from animals of each genotype evoked by flashes of three indicated intensities representing scotopic, mesopic, and photopic light intensity ranges

The data shown in Fig. 5 demonstrate that PA28α overexpression resulted in a robust improvement of retinal visual function in P23H mice. By the age of 3 months, untreated P23H mice experienced a roughly 2-fold reduction in the maximal amplitudes of a- and b-waves recorded across a broad range of light intensities. The overexpression of PA28α counteracted this reduction, yielding maximal a- and b-wave amplitudes ~40% and ~25% higher than observed in control P23H littermates (Fig. 5a). An even larger therapeutic effect was observed at 6 months. At this age, maximal amplitudes of both a- and b-waves in PA28α overexpressing mice were ~240% higher than in P23H control (Fig. 5b).

Perhaps the most foretelling data were obtained at dim flash intensities under ~0.05 cd/s/m^2^ at which ERG responses are driven almost exclusively by rods^39,40^. Under these conditions, light responses of 6-month-old P23H mice were essentially abolished, consistent with nearly all rods degenerating by this age. In contrast, P23H mice overexpressing PA28α retained an ability to produce robust light responses of nearly half normal amplitude. This result is particularly well-illustrated in a representative response to a flash of 0.005 cd/s/m^2^ (Fig. 5b, right panel), indicating that the functional impact on rod preservation upon PA28α overexpression is even more striking than the total nuclear count in Fig. 4 may suggest.

Also consistent with morphological data, PSMD11 overexpression resulted in a marginal preservation of retinal activity. In this case, statistically significant amplitude increase was observed only for b-waves, likely reflecting amplification of more subtle a-wave changes (Supplementary Fig. 8). Whereas this lesser therapeutic effect of overexpressing PSMD11 than PA28α may be potentially attributed to the difference in their relative cellular levels, the authors favor a possibility that it reflects the difference in the mechanisms by which each overexpression modifies the relationship between 20S proteasomes and their regulatory caps. Conceptually, these data are significant because they show that proteasomal activation improves the health of degenerating photoreceptors regardless of whether it is achieved by providing 20S cores with additional 11S caps or modifying 20S–19S interactions.

### Proteasome activity enhancement improves the survival of *Gγ*_*1*_^*−/−*^ rods

In another set of experiments, we tested whether an enhancement of proteasomal activity could delay the course of retinal degeneration in another mouse model, the knockout of the γ-subunit of photoreceptor-specific G protein transducin (the *Gγ*_*1*_^*−/−*^ mouse); this mouse served as a primary model in our previous study^9^. Rods of *Gγ*_*1*_^*−/−*^ mice produce large amounts of transducin β-subunit (Gβ_1_). Being unable to properly fold without its cognate Gγ_1_ partner, Gβ_1_ is continuously targeted for proteasomal degradation resulting in a proteostatic crisis and, ultimately, photoreceptor cell death^9^.

**Fig. 6:**
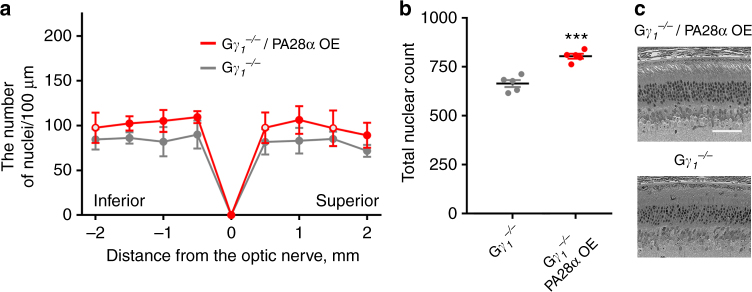
Overexpression of PA28α improves photoreceptor survival in *Gγ*_*1*_^*−/−*^ mice. **a** Spider diagrams representing the number of photoreceptor nuclei in 100 μm segments of the inferior and superior retina at various distances from the optic nerve head. Data were collected from five mice at 3 months of age and shown as mean ± SD. Data points for which the difference in the number of nuclei between treated and untreated animals was statistically significant (*p* < 0.05) are shown as filled circles and those for which statistical significance was not achieved as open circles. **b** The total number of nuclei in all eight 100 μm retinal segments represented in spider diagrams of **a**. Data are shown as mean ± SEM. **c** Representative images of inferior retina cross-sections at the 1 mm distance from the optic nerve head; scale bar: 50 μm. Mouse genotypes are indicted above the panels. See Supplementary Fig. 9 for images of representative cross-sections through the entire retinas

*Gγ*_*1*_^*−/−*^ mice were crossed with PA28α overexpressing mice and retinal morphology of the progeny was analyzed at the age of 3 months (Fig. 6 and Supplementary Fig. 9). The spider diagram in Fig. 6a shows that PA28α overexpression improved the photoreceptor survival across the entire retina. Integrating the nuclear counts for all locations analyzed in the spidergrams (Fig. 6b) revealed that overexpressing retinas contained 21% more photoreceptor nuclei than untreated *Gγ*_*1*_^−*/−*^ controls (*p* = 0.0002). Not only the number of surviving photoreceptors was higher, but also the morphological appearance of their nuclei was more uniform across the entire photoreceptor population (Fig. 6c).

However, the extent of photoreceptor rescue achieved upon PA28α overexpression (Fig. 6) was smaller than in P23H mice (Fig. 4). This result is not immediately intuitive because the degree of proteasomal insufficiency in *Gγ*_*1*_^*−/−*^ mice, judged from the degree of intracellular accumulation of the Ub^G76V^–GFP reporter, appears to be more pronounced than in P23H mice (Supplementary Fig. 4). A likely explanation for this discrepancy derives from the fact that Ub^G76V^–GFP accumulation reflects insufficient photoreceptor capacity to process ubiquitinated protein substrates, whereas PA28α overexpression has no significant effect on the ubiquitination-dependent protein degradation (Fig. 3). Accordingly, the degree by which photoreceptors may benefit from PA28α overexpression may not directly correspond to the degree of reporter accumulation.

**Fig. 7:**
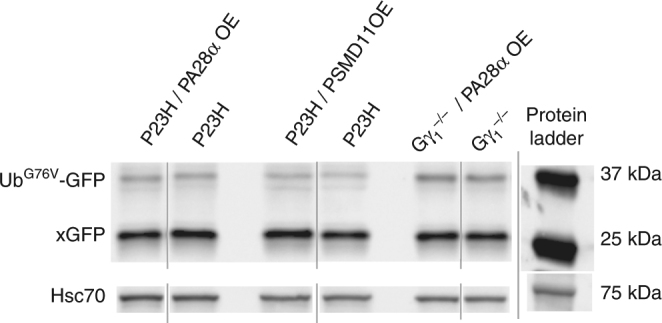
Overexpression of PA28α or PSMD11 does not affect accumulation of the Ub^G76V^-GFP reporter. The Ub^G76V^-GFP reporter was detected in retinal lysates from 1-month-old mice of indicated genotypes (30 μg total protein/lane) using an anti-GFP antibody; Hsc-70 was used as a loading control. The band representing the non-proteolyzed non-fluorescent GFP product co-accumulating with this reporter in cells suffering from proteasomal insufficiency^10,58^ is labeled as xGFP. Data are taken from one of the four similar experiments

This explanation would further predict that accumulation of the Ub^G76V^–GFP reporter may not directly correlate with the therapeutic effect achieved by PA28α overexpression. We tested this prediction in the last set of experiments by analyzing P23H and *Gγ*_*1*_^*−/−*^ mice expressing both PA28α and Ub^G76V^–GFP. As shown in Fig. 7, overexpression of PA28α was not accompanied by any notable reduction in Ub^G76V^–GFP accumulation in either model, which is consistent with the importance of ubiquitin-independent protein degradation for rescue using this approach.

## Discussion

Following initial demonstrations that insufficient activity of the ubiquitin–proteasome system is a prominent stress factor in multiple forms of inherited retinal degeneration^9,10^, we investigated whether ameliorating this stress by increasing the activity of photoreceptor proteasomes could have a therapeutic effect. Our most successful approach was to overexpress the PA28α subunit of the 11S proteasome cap. PA28α overexpression supported photoreceptor survival in degenerating retinas, with a particularly striking effect achieved in a common model of RP, the P23H mouse. While untreated mice lose nearly all rods by 6 months of age, PA28α overexpression preserves enough rods to sustain robust retinal visual outputs in dim light conditions.

The major impact of this study is that it provides a proof-of-principle that cellular proteostasis as a whole and proteasomes in particular could serve as a therapeutic target for treating RP and related diseases. The hallmark of RP is that the underlying mutations typically affect rods, whereas debilitating visual loss does not occur until rod degeneration reaches its final stages when otherwise healthy cones start degenerating as well. Accordingly, the period between the onset of rod degeneration and the patient becoming legally blind often spans several decades. In this context, the effect of PA28α overexpression in P23H mice, and even the relatively modest effect observed upon PSMD11 overexpression in these mice or PA28α overexpression in *Gγ*_*1*_^*−/−*^ mice, would correspond to years-to-decades of prolonged vision in patients.

The therapeutic effect of PA28α overexpression was achieved through the stimulation of ubiquitin-independent protein degradation. Lysates obtained from transgenic retinas displayed a significant increase in the level of ubiquitin-independent proteasomal activity, which we estimated to represent an ~40% activity increase in overexpressing rod photoreceptors. In contrast, the degradation of ubiquitinated substrates by the same lysates was not significantly different from WT controls, at least within the resolution capacity of available techniques. Consistent with the latter, the overexpression of PA28α in P23H or *Gγ*_*1*_^*−/−*^ rods was not associated with a detectable change in the intracellular accumulation of the Ub^G76V^-GFP reporter.

Another, closely related question is whether PA28α overexpression in photoreceptors leads to the formation of any hybrid proteasomes, containing 19S and 11S caps on the opposite sides of a 20S core particle^29,41^, or 20S cores singly or doubly capped by 11S. The intrinsic instability of proteasome complexes containing 11S caps formed exclusively by PA28α^20^ limits the experimental options to address this question. One approach was to analyze the ATP dependency of Suc-LLVY-amc degradation because hybrid proteasomes, but not 11S–20S complexes, rely on ATP for their stability. Our measurements revealed both ATP-dependent and ATP-independent components of the proteasome activity increase in the lysates from overexpressing retinas, suggesting that both types of proteasome complexes were present. Gel-filtration experiments were less foretelling. They revealed a high molecular mass shift in the elution profile of a small portion of overexpressed PA28α. Some of this shifted PA28α co-eluted with 20S cores and none with 19S caps. While consistent with the presence of 11S–20S complexes, this result does not entirely exclude that hybrid proteasomes were initially present in the retinal lysate but lost their 11S caps during the chromatography run due to the low affinity of homomeric 11S caps^20,24^. But regardless of whether or not hybrid proteasomes were formed along with the 11S–20S complexes, PA28α overexpression did not facilitate the processing of polyubiquitinated substrates in retinal lysates or decreased the Ub^G76V^–GFP accumulation in overexpressing photoreceptors.

Looking forward, this study lays the foundation for several exciting mechanistic directions. It will be important to understand why stimulation of ubiquitin-independent proteasomal degradation in degenerating photoreceptors improves their health. One potential explanation relates to the ability of proteasomes to degrade certain proteins without their polyubiquitination^42^. In many cases, this process still requires the involvement of 19S caps. However, there are two notable exceptions—the adaptive immune response and adaptation to oxidative stress—in which protein degradation is performed by 20S proteasomes themselves^43^. Oxidative stress is among the most frequently mentioned stress factors in RP pathology^3^, which makes it possible that increased 20S accessibility to oxidized substrates achieved through the 11S caps formed upon PA28α overexpression explains, at least in part, the therapeutic effect revealed in our study.

It would also be interesting to explore why this approach benefits P23H mice more than *Gγ*_*1*_^−/−^ mice. Conversely, it would be important to elucidate whether stimulation of ubiquitin-dependent protein degradation, for example by inhibiting substrate deubiquitination by the UPS14 subunit of the 19S cap^44^, would have a similar effect. We anticipate that addressing these mechanistic questions in future studies would allow better identification of patient cohorts that would most benefit from this approach.

Another experimental area, likely to be stimulated by our demonstration that retinal degeneration could be slowed down by an enhancement of cellular proteostasis, is to test whether a similar therapeutic effect could be achieved by inhibiting the rate of protein synthesis. This idea was recently put forward in a study employing pharmacological inhibition of protein translation in P23H rodent models^45^. While this treatment did improve the folding and trafficking of mutant rhodopsin, it ultimately accelerated photoreceptor cell death, apparently because the stabilized P23H mutant was toxic. Although this result was disappointing, it may be worthwhile to consider this strategy for alternative RP-causing mutations before generalizing this negative outcome.

Finally, our study places inherited retinal degenerations among the expanding group of neurodegenerative disorders whose progression could potentially be ameliorated by augmenting cellular proteostasis (reviewed in refs. ^46–48^). As an organ well-accessible to a broad array of interventions, from conventional small molecule treatments to gene therapy, the eye could serve as a nearly unmatched system to test the efficacy of any future therapy targeting cellular proteostasis in neurodegeneration. Furthermore, the outcomes of these treatments can be monitored with unparalleled quantitative precision using modern imaging techniques^49^. The early diagnosis of RP allows therapeutic modulation of the ubiquitin–proteasome system to begin early, thereby facilitating its validation and maximizing its gains.

## Methods

### Knockout and transgenic animals

Mouse care and experiments were performed in accordance with procedures approved by the Institutional Animal Care and Use Committee of Duke University. *Gγ*_*1*_^*−/−*^ mice were licensed from Deltagen Inc. (San Mateo, CA)and characterized previously^9^. P23H mice described in ref. ^35^ were purchased from Jackson Labs (stock #017628). Transgenic mice heterozygously expressing the Ub^G76V^-GFP reporter are described in ref. ^50^. Transgenic mice overexpressing PA28α and PSMD11 under the control of rhodopsin promoter were generated by Duke Transgenic Facility using standard protocols described in refs. ^40,51^ on the C57BL/6J background. Transgenic mouse lines were maintained by heterozygous breeding with C57BL/6J WT mice (stock #000664) and tested for the lack of Rd1 and Rd8 mutations. All experiments with overexpressing mice were performed using WT and/or non-expressing littermate controls; all animals in this study were pigmented; mice of both sexes were used.

### Antibodies

Rabbit antibodies against Gγ_1_ (SC-373; 1:10,000) and the β1 subunit of the 20S proteasome (SC-67345; 1:5000), as well as mouse antibody against β-actin (SC-47778; 1:5000), were from Santa Cruz Biotechnology. Rabbit polyclonal antibodies against the regulatory subunit 1, PSMD1, of the 19S cap (140682; 1:2000) and against the β5 subunit of the 20S core (3330; 1:2000) were from Abcam. Mouse monoclonal anti-eGFP antibody (632381; 1:5000) was from Clontech. Rabbit antibodies against PA28α (BML-PW8185; 1:5000) and HSC70/HSP73 (ADI-SPA-819; 1:10,000) were from Enzo Life Science. Rabbit antibody against PA28β (NB120-2940; 1:5000) was from Novus Biologicals. Mouse antibody against PSMD11 (1:2000) was from Abnova. Rabbit antibody against polyubiquitin (PA1-187; 1:4000) was from Thermo Fisher. Sheep anti-phosducin antibody (1:10,000) is described in ref. ^52^. Mouse monoclonal anti-rhodopsin antibody 4D2 (1:5000) was a gift from R.S. Molday (University of British Columbia). Secondary goat or donkey antibodies for western blotting conjugated with Alexa Fluor 680 (A-21057) and 800 (SA5-10044) (both 1:10,000), were from Invitrogen, secondary peroxidase-conjugated anti-rabbit (711-035-152) or anti mouse (715-035-150) antibodies (both 1:10,000) were from Jackson ImmunoResearch Laboratories. Protein bands were visualized and quantified using the Odyssey Infrared Imaging System (LI-COR Biosciences) and ChemiDoc Imaging System (BioRad). The photoreceptor outer segments were visualized by wheat germ agglutinin (WGA) 594 conjugate (W11262; 1:500) from Invitrogen. Uncropped gels used to generate images in the figures are shown in Supplementary Fig. 10.

### Size-exclusion chromatography

Retinal lysates obtained from two mice were cleared of debris by centrifugation (20 min at 18,000×*g* at 4 °C) and subjected to gel filtration on a Superose-6 attached to the FPLC system (Pharmacia) (Fig. 1 only) or a Superose-6 Increase column attached to the AKTA pure 25 system (GE Healthcare) at a flow rate of 500 μl/min in the running buffer containing 50 mM Tris-HCl (pH 7.4), 40 mM NaCl, 5 mM MgCl_2_, 10% glycerol, 1 mM DTT, and 1 mM ATP; 0.5 ml fractions were collected and proteins were precipitated by a 5-fold dilution in cold acetone at −20 °C overnight. Precipitated proteins were collected by centrifugation (15 min at 4 °C at 17,000×g), the pellets were air dried and solubilized in the SDS/PAGE sample buffer for western blotting. Bands were visualized and quantified using the Odyssey infrared imaging system (LiCor Bioscience).

### Native gel electrophoresis

All procedures, including tissue extraction, protein separation, and detection, were performed as described in ref. ^53^ without modifications. The 26S proteasome standard (A1200) was obtained from UbpBio.

### Quantitative mass spectrometry

The stoichiometric ratio of proteasomal subunits in retinal lysates from WT mice was determined by quantitative mass spectrometry using cold isotope-labeled peptides standards, as described in ref. ^9^. Synthetic peptide standards containing Lys (13C6, 15N2) or Arg (13C6, 15N4) were obtained from JPT Peptide Technologies (Berlin, Germany). The 20S core was represented by two peptides from the α7 subunit (LTVEDPVTVEYITR and ALLEVVQSGGK) and two peptides from the β1 subunit (GAVYSFDPVGSYQR and DVYTGDALR). The 11S cap was represented by two peptides from PA28α (TENLLGSYFPK and IEDGNNFGVAVQEK). The 19S cap was represented by two peptides from the PSMD5 subunit (LEAPLEELR and ELTGEDVLVR) and two peptides from the PSMD6 subunit (GAEILEVLHSLPAVR and FLLSLPEHR). Standards were selected based on their robust identification in a preliminary LC/MS/MS analysis of the whole retinal lysate, considering their ionization strength and chemical stability. To reduce peptides’ non-specific binding to test tubes, all dilutions were performed in 2% acetonitrile/0.1% formic acid containing 0.5 μM leucine enkephalin. In a typical experiment, mouse retinas were lysed in PBS buffer containing 1% Triton X-100 and cOmplete protease inhibitor cocktail (Roche); 40 μg of proteins from the lysate were separated by SDS-PAGE on a 10% gel. A set of 2–3 gel bands corresponding to the location of each targeted proteasomal subunit was spiked with of the corresponding peptide standard (100 fmol for α7 and β1 or 50 fmol for other subunits). Proteins were in-gel digested with trypsin and peptide mixtures were analyzed on a 75 μm × 150 mm BEH100 1.7 μm C18 column coupled to the Synapt G2 mass spectrometer (Waters). LC/MS analysis was performed for each sample using a 90-min gradient of acetonitrile (6–32%) in 0.1% formic acid. Peptide assignments in ion chromatograms were based on precise co-elution with standard peptides, considering the corresponding mass shifts. Peptide ion peaks for the first isotope in the cluster were integrated using MassLynx 4.1 software (Waters) and the peptide amounts were calculated from the ratios between the peak areas of peptides from retinal lysates and standard peptide. Each MS analysis was performed in duplicate for three independently obtained mouse lysates. To obtain a stoichiometric ratio among the 20S, 19S, and 11S components, data were averaged across all lysates and MS repeats. For the final calculation, we assumed that each 20S core contains two α7 and β1 subunits, each 19S cap contains one PSMD5 and PSMD6 subunit, and each 11S contains an average of 3.5 PA28α.

### Proteasome activity assays with fluorogenic peptides

Proteolytic activities of proteasomes in retinal lysates were measured using the fluorogenic 7-Amino-4-methylcoumarin (AMC)-conjugated peptide substrates following the protocols described in ref. ^15^. Both retinas from one mouse were homogenized on ice using a 2 ml Dounce homogenizer in 400 μl buffer containing 50 mM Tris-HCl (pH 7.6), 40 mM KCl, 5 mM, MgCl_2_, 1 mM DTT, 10% glycerol, and 5 mM ATP in the presence of cOmplete™ Protease Inhibitor Cocktail (Sigma-Aldrich), followed by sedimentation for 20 min at 18,000×*g*. The time course of AMC fluorescence increase was monitored in 50 μl aliquots for 2 h at 37 °C using a SpectraMax M5 (Molecular Devices) or CLARIOstar (BMG Labtech) plate readers with the following peptides: 100 μM Suc-LLVY-amc (a substrate for the chymotrypsin-like proteasomal activity; UbpBio), 50 μM Boc-LRR-amc (a substrate for the trypsin-like activity; UbpBio), and 250 μM Z-LLE-amc (a substrate for the caspase–like activity; UbpBio). The absolute amounts of proteolyzed substrates were measured using the AMC standard (Sigma-Aldrich). To study the ATP dependency of proteasomal activity, aliquots of retinal extracts were incubated with apyrase (Sigma) at the concentration of 10 units/ml for 30 min at room temperature in order to hydrolyze the endogenous ATP, and the measurements were repeated as above in the absence of added ATP.

### Proteasome activity assays with polyubiquitinated substrates

26S proteasome activity was assessed by monitoring the degradation of ubiquitinated peptide and protein substrates. The ubiquitinated peptide substrate was prepared as previously described^54^. Protein substrate was generated by in vitro polyubiquitination of truncated human WW domain-binding protein 2 (WBP2) followed by the labeling reaction using Alexa Fluor 488 C5 Maleimide (Thermo Fisher Scientific). Retina lysate was prepared in the buffer containing 40 mM HEPES (pH7.6), 100 mM KCl, 5 mM MgCl_2_, 2 mM ATP, 1 mM DTT in the presence of cOmplete™ Protease Inhibitor Cocktail (Sigma-Aldrich). Fluorescence polarization assay was performed in the same buffer supplemented with 2 μM ubiquitin aldehyde (Boston Biochem). The reaction was initiated by mixing 4 μl retinal lysate containing 1.5 mg/ml protein with 11 μl fluorescent-labeled peptide (5 nM) or protein (2.5 nM) substrate. Assays were performed in low-volume 96-well solid black plates (Molecular Devices) in triplicate. The reaction was monitored at 30 °C using a PHERAstar plate reader (BMGlabtech) with excitation at 480 nm and emission at 520 nm.

### Histological techniques

Agarose-embedded retinal cross-sections were prepared as described^55^, collected in 24-well plates, and incubated for 2 h with Alexa Fluor 594 conjugate of wheat germ agglutinin (Invitrogen) in PBS containing 0.1% Triton X-100. Sections were washed three times in PBS, mounted with Fluoromount G (Electron Microscopy Sciences) under glass coverslips, and visualized using a Nikon Eclipse 90i confocal microscope. GFP fluorescence was excited at 488 nm. Plastic-embedded retinal cross-sections (1 μm) were prepared as described in refs. ^9,56^ and stained with toluidine blue for light microscopy. Nuclear count in 100 μm segments of the outer nuclear layer was performed in sections cut through the optic nerve at 500-μm steps from the optic nerve head. Tangential sectioning of flat-mounted frozen retinas was performed as described in refs. ^22,56^.

### Electroretinograms (ERGs)

ERGs were recorded from dark-adapted mice as described in refs. ^40,57^ using the Espion E2 system with a ColorDome ganzfeld stimulator (Diagnosys LLC, Littleton, MA). Mice were anesthetized by an intraperitoneal injection of 90 mg/kg ketamine and 9 mg/kg xylazine. Pupils were dilated with a mixture of 1% cyclopentolate-HCl and 2.5% phenylephrine. Eyes were kept lubricated during the recordings by a 1% carboxymethylellulose sodium gel. Body temperature was maintained by a heated platform. Simultaneous recording were made from both eyes using gold contact lens electrodes (Mayo Corporation, Oasuka, Japan), with stainless steel needle electrodes (Ocuscience) in the mouth (reference) and at the base of the tail (ground). ERG signals were sampled at 1 kHz and recorded with 0.15 Hz low frequency and 500 Hz high frequency cut-offs. Responses to flashes from 0.00025 cd/s/m^2^ to 500 cd/s/m^2^ with averaged trials of 15 to 1 and inter-flash intervals of 5 s to 180 s. Data points from the a-wave and b-wave stimulus-response curves were fitted by single or double hyperbolic functions, respectively, using the least-square fitting procedure. *p*-Values were calculated to determine the statistical significance between the response amplitudes in P23H mice overexpressing PA28α or PSMD11 and control P23H mice at each flash intensity using independent two-tailed *t*-tests in Prism software (version 7.03).

### Statistical methods

Data were analyzed using the Prizm GraphPad software. *p*-Values were calculated using two-tailed homoscedastic *t*-test. Data are presented as mean ± SEM, except for the spidergrams where data are presented as mean ± SD. *p*-Values on the figures are indicated as follows: *p < 0.05, **p < 0.01, ***p < 0.001, ****p < 0.0001, and n.s. for *p* > 0.05. No statistical methods were used to pre-determine sample sizes, but sample sizes are similar to those used in previous published studies. No data were excluded from the analysis. No randomization was used and animal genotypes were not blinded.

### Data availability

All relevant data are available on reasonable request from the authors.

## Electronic supplementary material

Supplementary Figures
