# Supplementary material for: Increased proteasomal activity supports photoreceptor survival in inherited retinal degeneration
Source: Nat Commun. 2018 Apr 30;9:1738. doi: 10.1038/s41467-018-04117-8 (PMC5928105; doi:10.1038/s41467-018-04117-8)
Supplement: Supplementary file 1 — Supplementary Figures [file 41467_2018_4117_MOESM1_ESM.pdf]

Increased proteasomal activity supports photoreceptor survival in inherited retinal degeneration

Lobanova *et al*.

Supplementary Figures

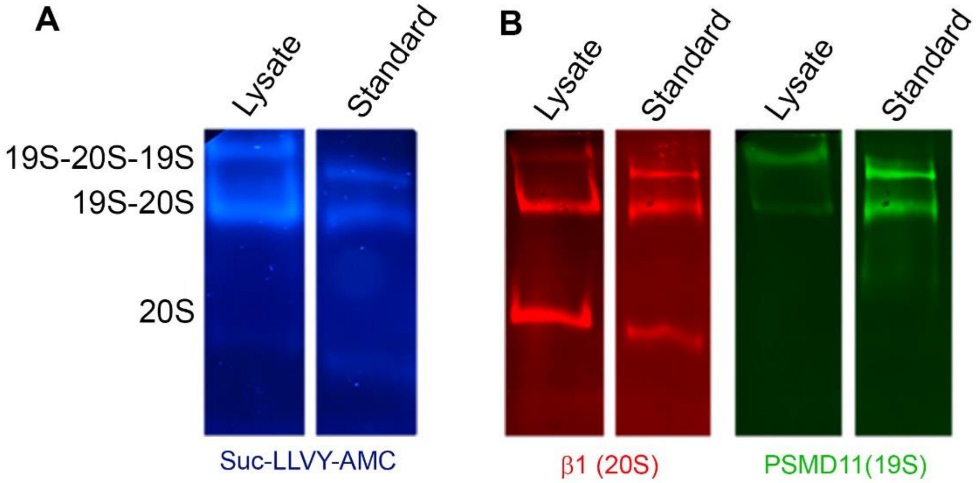

Supplementary Figure 1. Native gel electrophoresis of the retinal extract from a WT mouse. Samples containing the retinal extract (60 μg total protein) and 26S proteasome standard (5 μg) were analyzed side-by-side using native PAGE. Proteasomal complexes were visualized by detection of either (A) in-gel chymotrypsin-like activity of proteasomal complexes using the Suc-LLVY-amc substrate or (B) immunoblotting with antibodies against β1 subunit of the 20S core (red) and PSMD11 subunit of the 19S cap (green). The data are taken from one of three similar experiments.

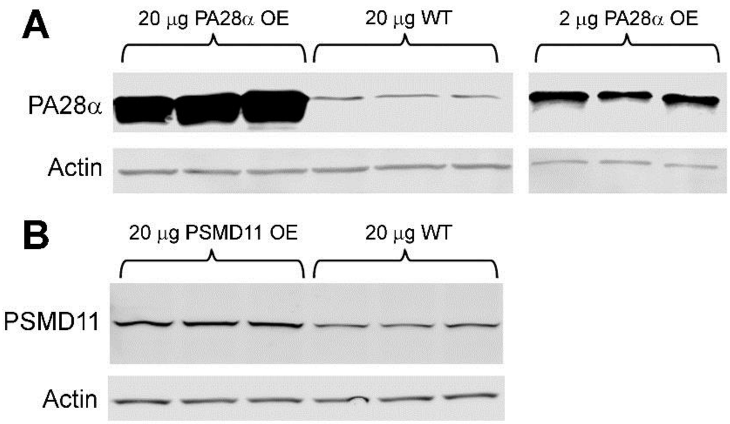

Supplementary Figure 2. Low variability in PA28α (A) and PSMD11 (B) overexpression across individual animals from the transgenic mouse lines analyzed in this study. Each protein was detected by Western blotting in retinal lysate aliquots from mice of indicated genotypes. The total protein amount in each sample is shown above the panels. β-actin was used as a loading control. Mice were 2 months old.

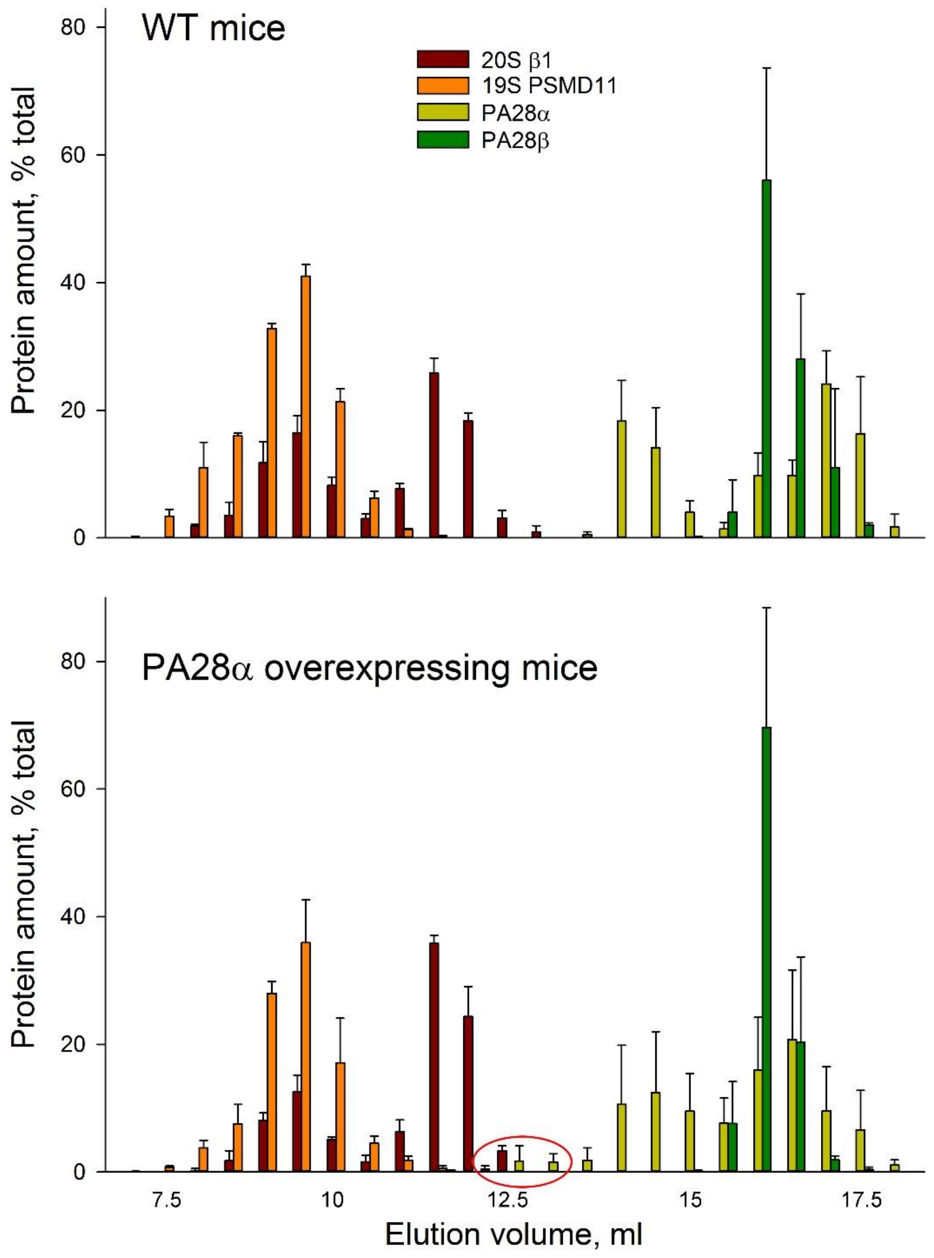

Supplementary Figure 3. Quantitative analysis of proteasome components' distributions across size-exclusion chromatography profiles from WT and PA28α overexpressing retinal lysates. Proteins from retinal lysates obtained from 2 mice (WT or PA28α overexpressing) were separated by size-exclusion chromatography on a Superose-6 Increase column. Proteins in 0.5 ml fractions were probed by Western blotting using antibodies against the β1 subunit of 20S proteasome core, PSMD11 subunit of 19S proteasome cap, PA28α subunit of 11S cap and PA28β subunit of 11S cap. Relative contents of each protein across chromatography fractions were quantified using the Odyssey infrared imaging system (LiCor Bioscience). The content of each protein across individual chromatography fractions is expressed as % of its total amount. The leftward shift in the PA28α profile is highlighted with a red oval. Data are averaged from three independent experiments and presented as mean ± SD.

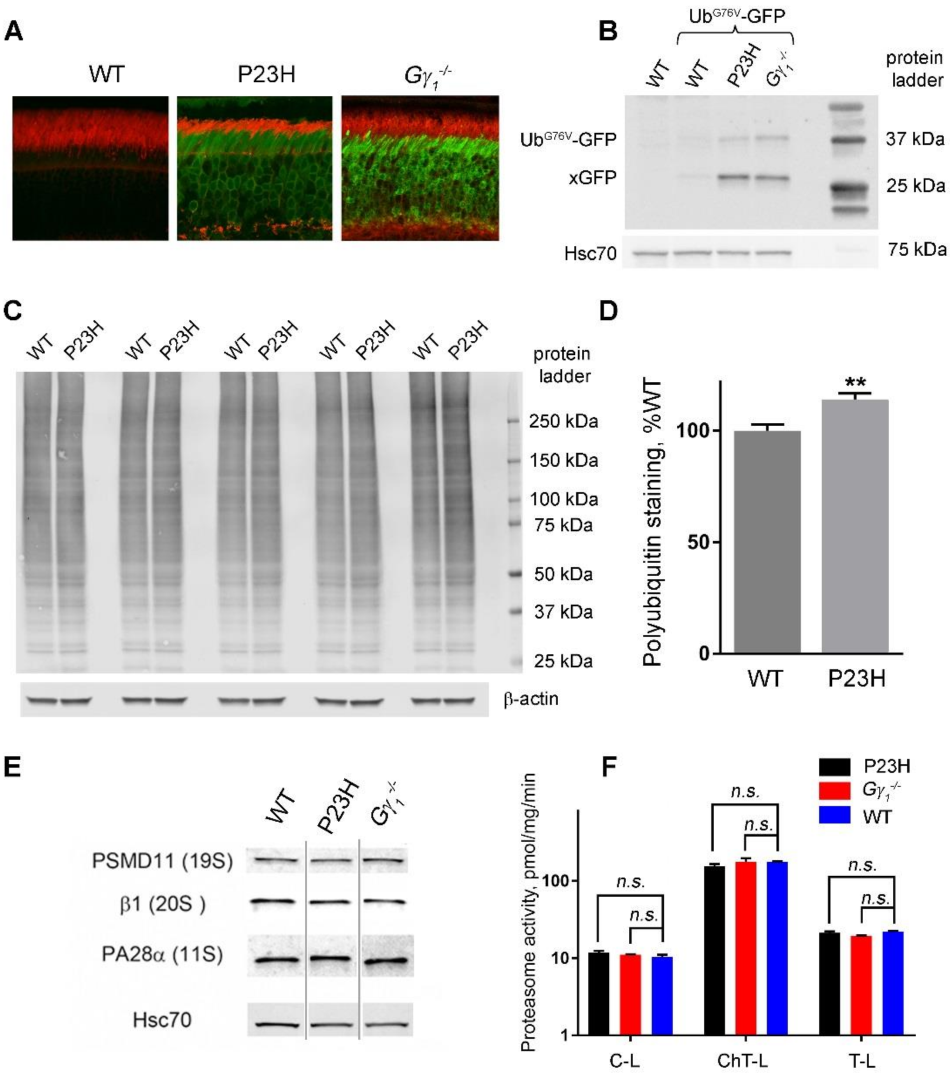

Supplementary Figure 4. Evidence of insufficient proteasomal capacity in P23H mice. (A) Ub^{G76V}-GFP reporter fluorescence (green) in retinal cross-sections of 1-month-old P23H mice. Data for previously characterized $G\gamma_1^{-/-}$ and WT mice are shown for comparison. Rod outer segments are stained with wheat germ agglutinin (WGA) conjugated to Alexa 594 (red). (B) Western blot detection of the Ub^{G76V}-GFP reporter in retinal lysates from 1-month-old mice of indicated genotypes using an anti-GFP antibody (15 μg protein/lane); Hsc-70 was used as a loading control. (C) Western blot immunostaining of polyubiquitin chains in the retinas of five pairs of P23H mice and their WT littermates (30 μg protein/lane); β-actin was used as a loading control; the analysis was conducted at P25. (D) Averaged values of the polyubiquitination signal densities in panel C (mean ± SEM; n=5). Data are normalized for the averaged density of WT measurements. (E) Western blot detection of representative proteasome subunits in retinal extracts from 1-months-old mice of indicated genotypes (30 μg protein/lane); Hsc-70 was used as a loading control. (F) Three enzymatic activities of the proteasome, chymotrypsin-like, trypsin-like and caspase-like, measured in retinal extracts from 1-month-old mice of indicated genotypes (mean ± SEM; n=3). In all panels representative data are taken from at least three independent experiments.

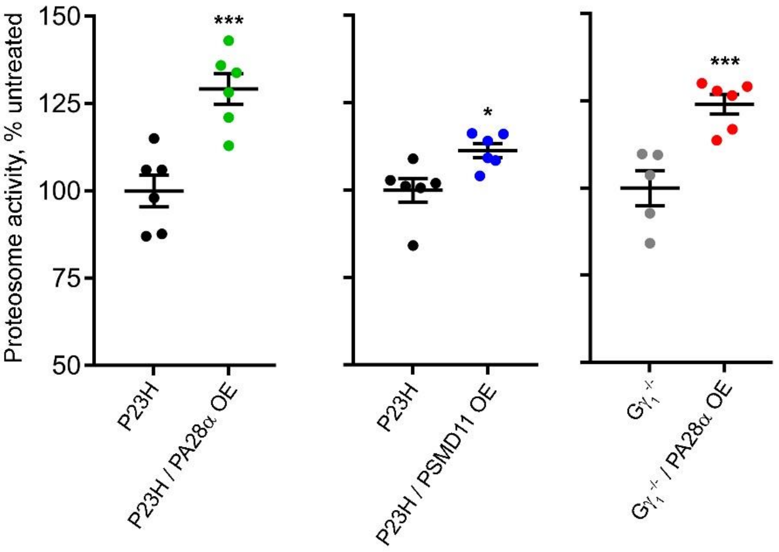

Supplementary Figure 5. Chymotrypsin-like proteasomal activity in retinal extracts from 1-month-old mice of indicated genotypes. Measurements were performed in the presence of ATP. The number of measurements was 5 for untreated $G\gamma_1^{-/-}$ mice and 6 for all other conditions. The data are shown as mean ± SEM.

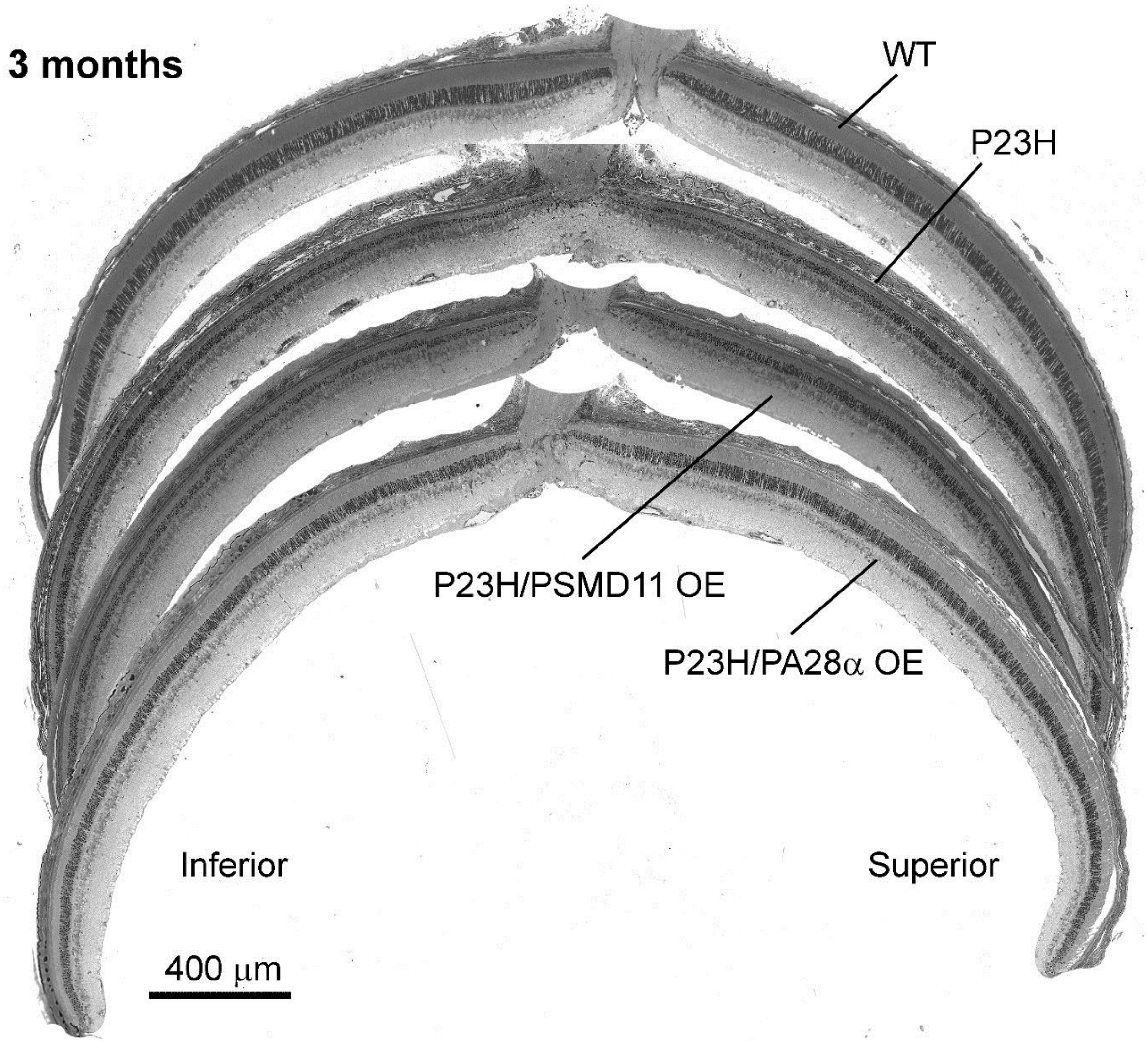

Supplementary Figure 6. Overexpression of PA28α or PSMD11 slows down photoreceptor loss in 3 months old P23H mice. Plastic sections cut through the entire mouse retinas of indicated genotypes were stained by toluidine blue (see Figure 4 for the number of mice analyzed from each genotype).

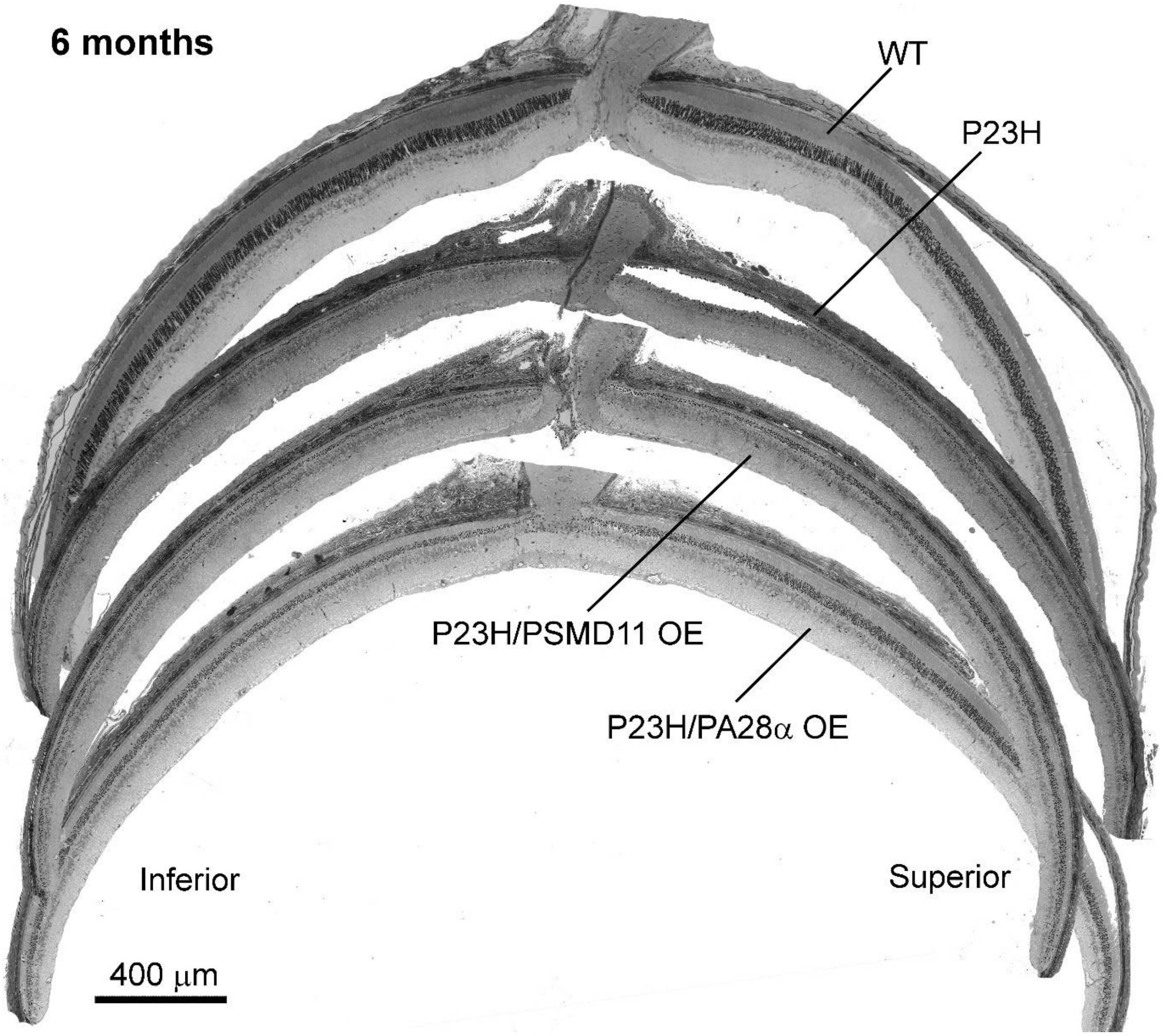

Supplementary Figure 7. Overexpression of PA28α or PSMD11 slows down photoreceptor loss in 6 months old P23H mice. Plastic sections cut through the entire mouse retinas of indicated genotypes were stained by toluidine blue (see Figure 4 for the number of mice analyzed from each genotype).

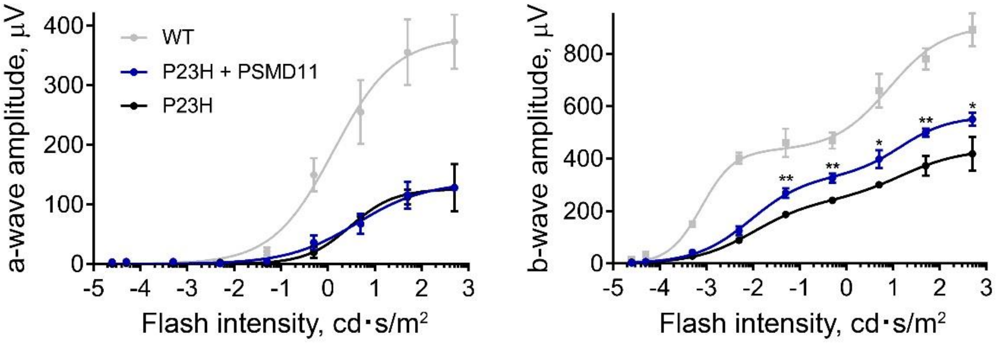

Supplementary Figure 8. ERG recordings from P23H mice overexpressing PSMD11. Response amplitudes of ERG a- and b-waves evoked by light flashes of increasing intensity were measured at the age of 3 months. Data are averaged from 4 independently recorded eyes and fitted using a single (for the a-wave) or double (for the b-wave) hyperbolic function. Error bars represent SEM.

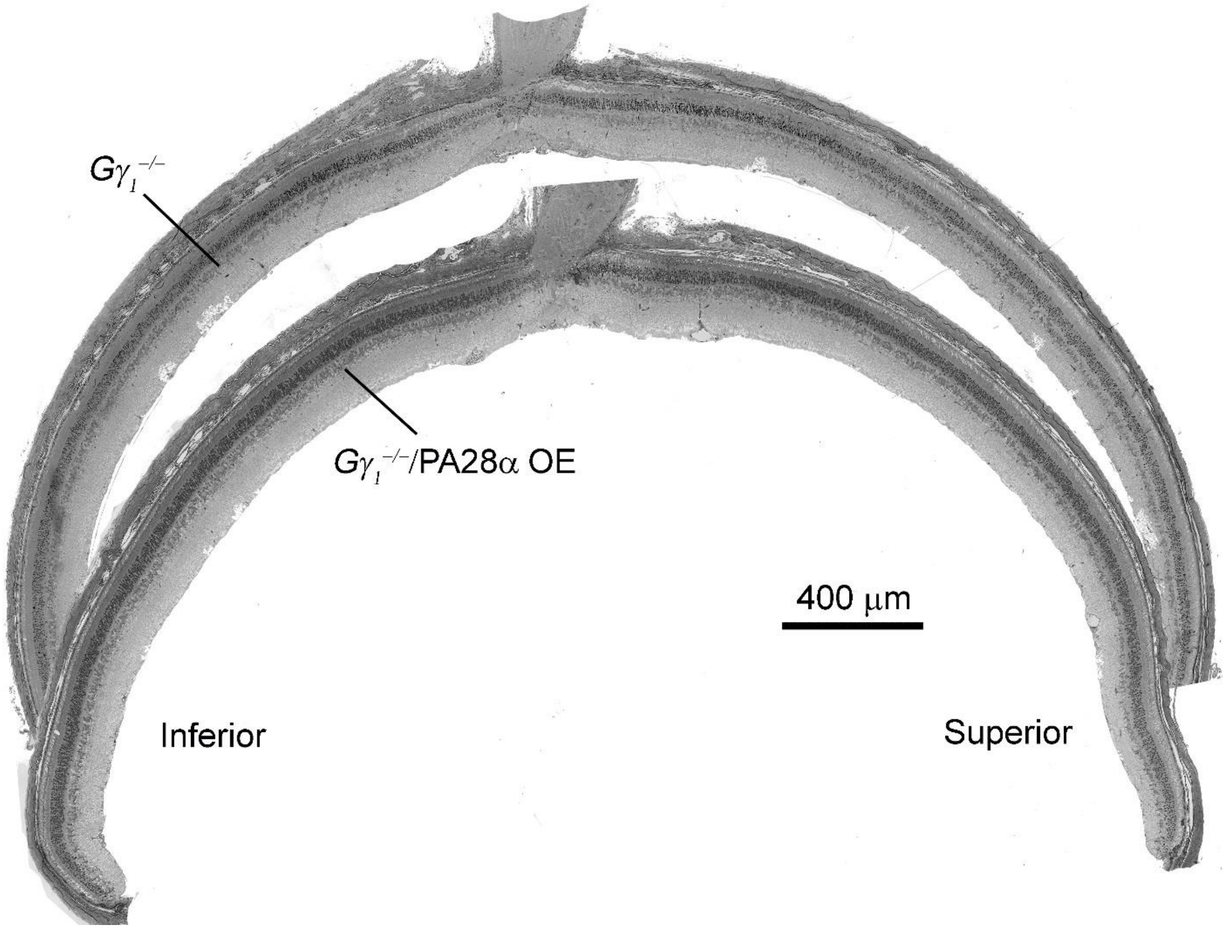

Supplementary Figure 9. Overexpression of PA28α slows down photoreceptor loss in the retinas of $G\gamma_1^{-/-}$ mice. Plastic sections cut through the entire retinas of 3-months-old mice of indicated genotypes were stained by toluidine blue. Images are taken from one of 5 mice of each genotype.

Blots for Figure 1B

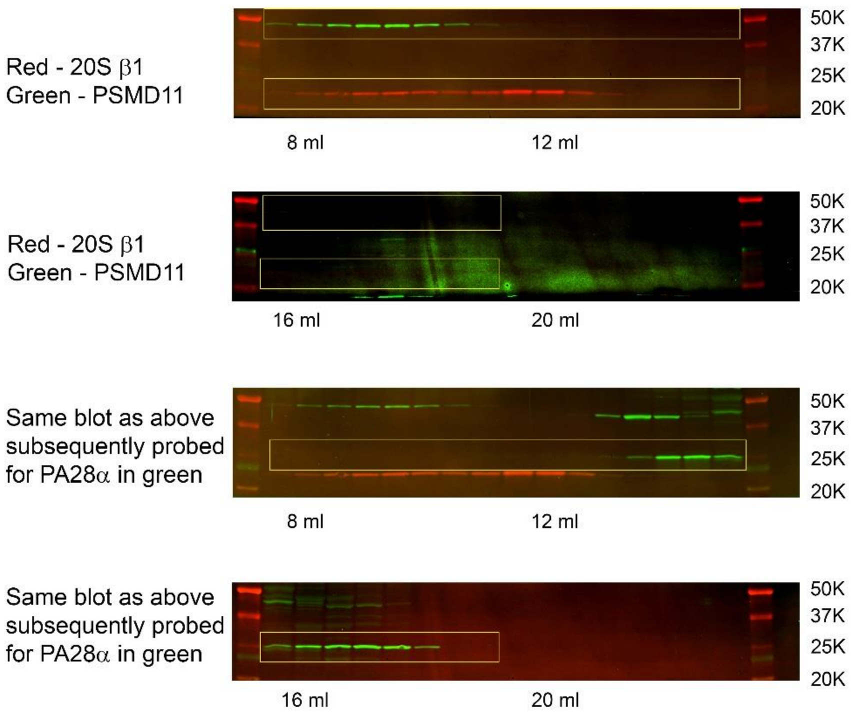

Blots for Figure 1C

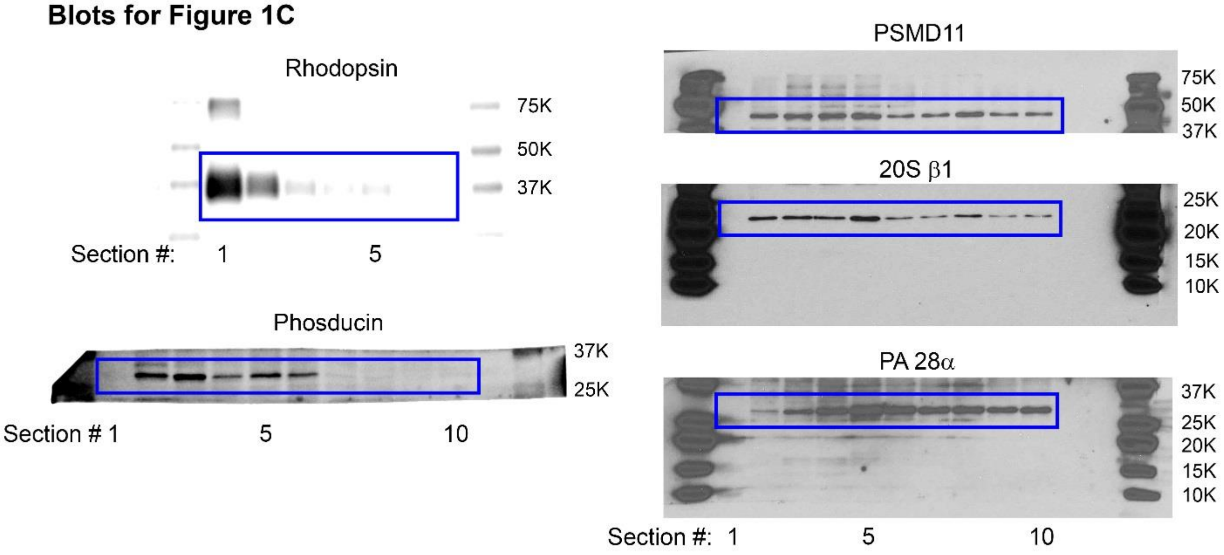

Blots for Figure 2D

WT; 20S β1:

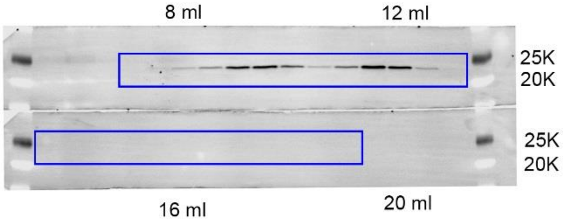

WT; PSMD11:

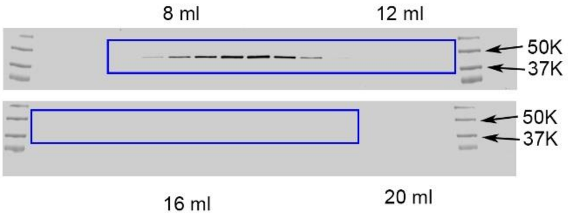

WT; PA28α:

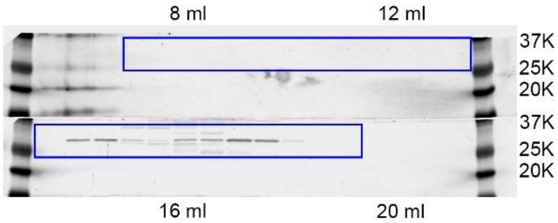

PA28α OE, 20S β1:

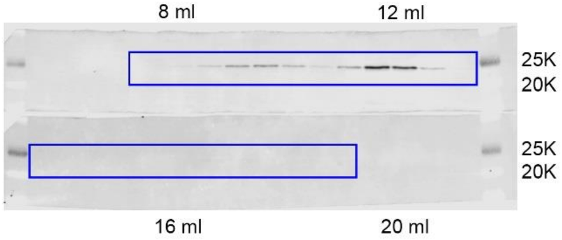

PA28α OE, PSMD11:

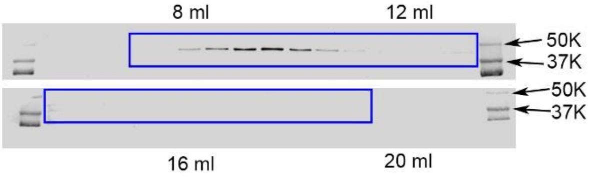

PA28α OE, PA28α:

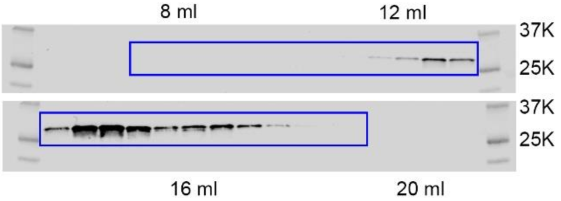

PSMD11 OE, 20S β1:

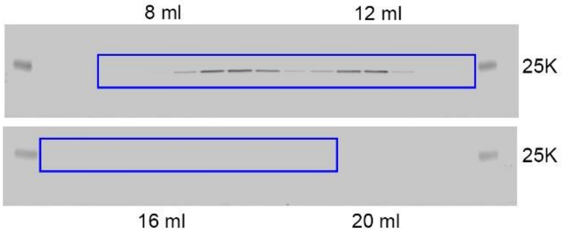

PSMD11 OE, PSMD11:

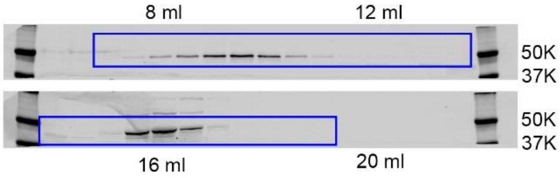

PSMD11 OE, PA28α:

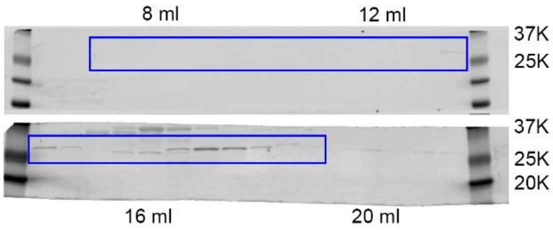

Blot for Figure 2A

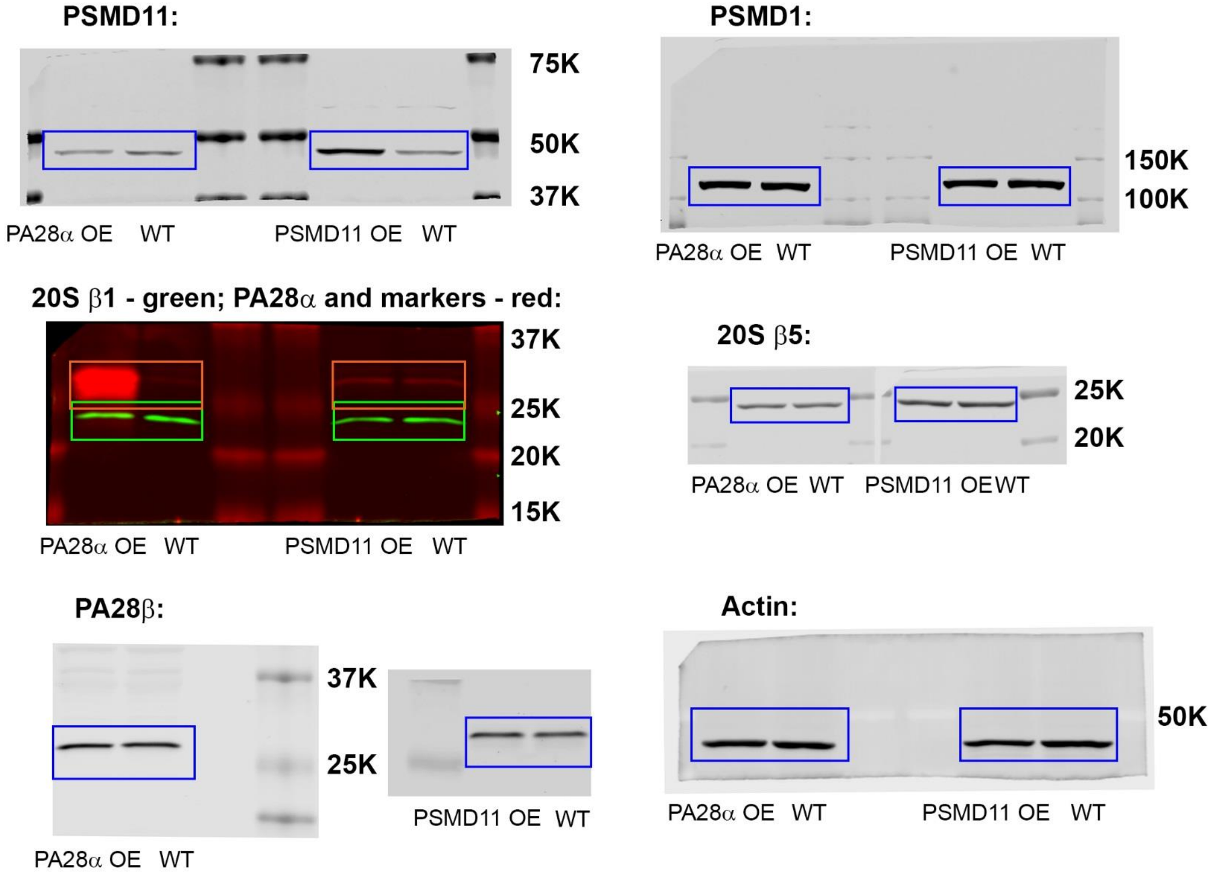

Blot for Figure 7

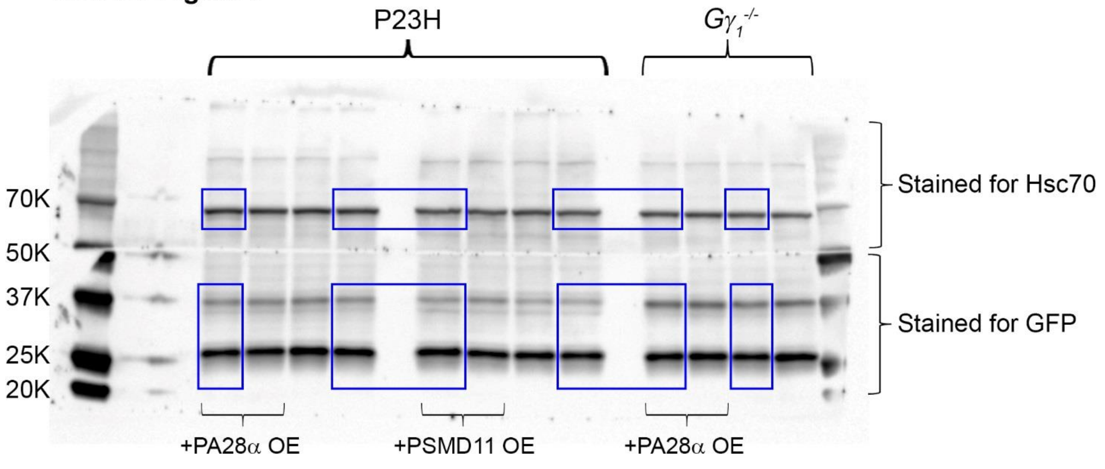

Supplementary Figure 10. Uncropped images of Western blot in Figures 1, 2 and 7.
